# Genome-wide association study and selection for field resistance to cassava root rot disease and productive traits

**DOI:** 10.1371/journal.pone.0270020

**Published:** 2022-06-16

**Authors:** Camila Santiago Hohenfeld, Adriana Rodrigues Passos, Hélio Wilson Lemos de Carvalho, Saulo Alves Santos de Oliveira, Eder Jorge de Oliveira

**Affiliations:** 1 Universidade Estadual de Feira de Santana, Feira de Santana, Bahia, Brazil; 2 Embrapa Tabuleiros Costeiros, Jardins, Aracaju, Sergipe, Brazil; 3 Embrapa Mandioca e Fruticultura, Cruz das Almas, Bahia, Brazil; Jeju National University, REPUBLIC OF KOREA

## Abstract

Cassava root rot disease is caused by a complex of soil-borne pathogens and has high economic impacts because it directly affects the tuberous roots, which are the main commercial product. This study aimed to evaluate cassava genotypes for resistance to root rot disease in a field with a previous history of high disease incidence. It also aimed to identify possible genomic regions associated with field resistance based on genome-wide association studies. A total of 148 genotypes from Embrapa Mandioca and Fruticultura were evaluated over two years, including improved materials and curated germplasms. Analysis of phenotypic data was conducted, as well as a genomic association analysis, based on the general linear model, mixed linear model, and fixed and random model circulating probability unification. The observed high disease index (ω) was directly correlated with genotype survival, affecting plant height, shoot yield, and fresh root yield. The genotypes were grouped into five clusters, which were classified according to level of root rot resistance (i.e., extremely susceptible, susceptible, moderately susceptible, moderately resistant, and resistant). The 10 genotypes with the best performance in the field were selected as potential progenitors for the development of segregating progenies. Estimates of genomic kinship between these genotypes ranged from -0.183 to 0.671. The genotypes BGM-1171 and BGM-1190 showed the lowest degree of kinship with the other selected sources of resistance. The genotypes BGM-0209, BGM-0398, and BGM-0659 showed negative kinship values with most elite varieties, while BGM-0659 presented negative kinship with all landraces. A genome-wide association analysis detected five significant single nucleotide polymorphisms related to defense mechanisms against biotic and abiotic stresses, with putative association with fresh root yield in soil infested with root rot pathogens. These findings can be utilized to develop molecular selection for root rot resistance in cassava.

## Introduction

Cassava (*Manihot esculenta* Crantz) is a semi-woody perennial shrub from the Euphorbiaceae family with upright or branched stems. It has a tuberous root system responsible for storing starch, an important source of carbohydrates. Globally, cassava is one of the most rapidly expanding staple crops due to its wide diversity of uses that allow its economic exploitation via direct use (i.e., consumption as food) or utilization in many industrial sectors, such as textiles, paper, food, and beverages [1]. According to the Food and Agriculture Organization (FAO) of the United Nations, global production of cassava amounted to 302.6 million tons on approximately 28.24 million hectares in 2020 [2]. Notably, Brazil is considered an important center of gender diversification [3], responsible for the production of 18.20 million tons of cassava root grown on approximately 1.21 million hectares. This makes Brazil the sixth-largest global producer of cassava, surpassed only by Nigeria, the Democratic Republic of the Congo, Thailand, Ghana, and Indonesia. Despite Brazil’s prominence in global cassava production, expansion of cassava planting faces obstacles associated with low fresh root yield compared to crop potential due to the use of unimproved varieties, the occurrence of abiotic stresses (i.e., drought) [4], and biotic stresses such as diseases caused by fungi, bacteria, and viruses [5].

Cassava root rot disease (CRRD) is one of the most destructive diseases for this crop due to the persistent nature of causal agents in the cultivation area, even in the absence of a host. The economic importance of CRRD has been increasing in the main cassava-producing countries because it causes a progressive reduction in yield, with reports of 37% average losses, even among improved varieties with some level of resistance [6]. Notably, losses can reach up to 100% when using susceptible varieties [7], making subsequent crop cycles nearly inviable or even unfit for consumption and/or processing [8, 9]. The CRRD complex can comprise different species of plant pathogens, and its symptoms differ based on the pathogen species involved [10]. In Brazil, dry root rot is usually caused by fungi belonging to the genus *Fusarium* and the species *Macrophomina pseudophaseolina*, whereas soft root rot is associated with oomycetes such as *Phytophthora* spp., *Pythium* spp., and *Phytopythium* spp. Black rot is mainly caused by species from the Botryosphareacea family, such as *Lasiodiplodia* spp. and *Neoscytalidium dimidiatum* [11–16].

Although cassava stems and leaves can also exhibit symptoms, the main affected tissue are the tuberous roots. This makes it difficult for producers to recognize the disease [17], since the aboveground parts of the plant can remain asymptomatic for long periods; in many cases, the disease is only identified at the time of harvest. Additionally, the cassava plant has a root system that may camouflage the effects of rotting, which hinders diagnosis and monitoring of epidemic development and management [18, 19].

The main strategy for root rot control involves the use of resistant varieties. This is considered the most effective management strategy due to its high cost-benefit ratio resulting from low environmental impact and easy adoption by producers [7, 10, 20, 21]. Furthermore, other integrated disease management practices remain incipient, such as the use of biological or chemical controls and even the induction of soil suppressiveness through the addition of organic matter [22]. However, the presence of different plant pathogen genera in the same crop area leads to the need to develop cassava varieties that are multi-resistant to the root rot complex [10, 16, 18, 19, 21].

The main problem related to management in the field is selection for resistance by breeding programs, which can be aggravated due to the presence of different genomic regions putatively associated with resistance to different species of pathogens that cause rot [23]. This makes identifying pathogens present in the area used for selection essential to the establishment of a generation of varieties with broad resistance. For example, the varieties *BRS* Kiriris and BRS Aramaris (sin = Cigana Preta) were selected in Northeastern Brazil as sources of resistance to root rot disease from fields with high inoculum pressure in the coastal plain region [24]. However, it remains unknown whether these varieties are resistant to all groups/species of pathogens or only to a specific group and/or species.

Screening in areas with a history of high disease pressure contributes significantly to the identification of parentals with good genetic resistance or even genotypes that can be promptly used in environments with a history of root rot disease [7, 21]. Although phenotyping in the field is costly, this approach facilitates more direct and consistent selection, since it is possible to evaluate a larger number of genotypes concomitantly, immediately exclude those that are highly susceptible, and perform selections based on the agronomic characteristics of interest for the target environments [25].

The objective of this study was to evaluate cassava genotypes—including curated germplasms, landraces, and improved hybrids—for root rot resistance by means of selection in a field with a history of the disease and high inoculum potential, based on the evaluation of two independent trials conducted over two years of evaluation (2014–2015 and 2016–2017). This study also aimed to identify putative genomic regions associated with field resistance and productive traits through genome-wide association studies (GWAS).

## Materials and methods

In this study, trials were conducted in Embrapa Tabuleiros Costeiros, an experimental field in Umbaúba, Sergipe, Brazil (11°22’37.9”S, 37°40’29.6”W, 109m). The soil of the experimental field has a sandy clay texture. The chemical and physical soil data are available in the S1 Table. The coastal plain region is characterized by the presence of a cohesive soil horizon, limiting water drainage, and a tropical monsoon climate with dry summers (type Am, according to Köppen-Geiger) [26]. A total of two (n = 2) trials were conducted during two growing seasons (2014–2015 and 2016–2017) in an area with a natural occurrence of cassava root rot pathogens. Climatic data was obtained from INMET [27], Brazil’s national meteorological institute, and the average for each season is shown in the S2 Table.

At the time of planting, the soil was tilled by plowing and harrowing, followed by furrowing along the planting lines. Planting holes were made along the furrows at a depth of 10 cm. Initial fertilization for each independent experiment was conducted based on the chemical analysis of the soil and the requirements of the cassava. The soil fertilization was performed using single superphosphate—Ca(H_2_PO_4_)_2_ + CaSO_4_.2H_2_O—200 g per linear meter on the furrows. For nitrogen and potassium, fertilizer was added by top dressing: urea (CH_4_N_2_O) and potassium chloride (KCl) in a 2:1 ratio, with 200 g applied per linear meter.

The planting used 20-cm-long cuttings, buried horizontally, spaced 0.9 m between rows, and spaced 0.6 m between plants. Both trials were harvested 10 months after planting, from August (2014 and 2016) to June (2015 and 2017). Supplementary water from a water truck was applied to the soil when necessary. Crop management followed the recommendations for cassava [28].

### Survey of root rot pathogens in the field

Before setting up the trials in 2014, 16 soil samples were collected from an area naturally infested with root rot, which were used to select sources of resistance based on successive cultivation of cassava for at least 10 years in the city of Umbaúba, State of Sergipe (11°22’37.9"S, 37°40’29.6"W). To obtain the isolates, the soil samples were distributed in transparent plastic boxes (gerbox) with 100 g of soil per box, and then baits were made from the deposition of disinfested cassava root fragments. After 4 days, the fragments with typical rot symptoms were cut into small pieces (0.5 cm), immersed for 1 minute in ethanol (70%), followed by 1 minute in sodium hypochlorite solution (5%), and subsequently washed with sterile distilled water three times. The fragments were placed to dry on sterilized filter paper and soon after, placed in potato-dextrose-agar (PDA) medium and incubated at 24°C for 7 days with a 12 h photoperiod.

The fungal isolates were given the pathogenicity test using the detached root methodology [20], in which the central region of each root was perforated (6 mm in diameter) and inoculated with discs of culture medium containing structures of the tested pathogens. Discs of PDA medium (without fungal growth) were used as a control with the same number of treatments. Assays were carried out in growth chambers with temperatures controlled at 26±2 °C, 12 h of light, and relative humidity at >85%. The roots were kept on autoclaved filter paper covered with transparent polyethylene bags to keep the environment moist. The evaluations were performed ten days after inoculation, with the injured area measured by means of digital analysis with the aid of the ImageTool Program (University of Texas Health Science Center, San Antonio, TX, USA).

For molecular identification, the cetyltrimethylammonium bromide (CTAB) method was used to extract DNA from the isolates. After extraction, the solution containing the DNA was stored at -20°C. The quality and amount of the total DNA was measured by visual comparison with the λ phage DNA at concentrations of 30, 50, and 100 ng running on a 1% agarose gel (80V.60min-1) stained with ethidium bromide (1.2 μl.100mL^-1^). Amplification of the internal transcribed spacer (ITS) region of the isolate rDNA was performed using the universal primers ITS1 (5’ TCC GTA GGT GAA CCT GCG G 3’) and ITS4 (5’ TCC TCC GCT TAT TGA TAT GC 3’). PCR reactions included an initial denaturation phase at 94°C for 2 minutes, followed by 35 composite cycles of denaturation at 94°C for 35 seconds, 1 minute annealing at 52°C and extension at 72°C for 1 minute, and a final extension phase at 72°C for 10 minutes. Amplification was performed in a VeritiTM 96 Well Thermal Cycler (Applied Biosystems, Foster City, CA, USA). The amplified fragments were visualized under ultraviolet light and photographed on Carestream Gel Logic 212 Pro equipment (Kodak, Rochester, NY, USA). The PCR reaction products were purified using commercial column purification kits (UltraClean^®^ PCR, Clean-Up Kit, Mobio Laboratories, Inc.) and then sent for sequencing at the Génome Québec company (Montréal, Québec, Canada) in two directions (forward and reverse). The generated consensus sequences were compared with those deposited in the National Center for Biotechnology Information (NCBI) database (website: http://www.ncbi.nlm.nih.gov), using the BLASTn tool to confirm the gender of each of the isolates.

### Phenotyping and genotyping panel

A total of 148 cassava genotypes, including curated germplasms belonging to the Banco Ativo de Germoplasma de Mandioca (BAG-Mandioca) of Embrapa Mandioca e Fruticultura (Cruz das Almas, Bahia), landrace varieties, and improved hybrids, were evaluated. The genotype sets were: four elite clones (9975–01, 16–07, 2002-01-01, and 9655–02), 11 improved varieties (BRS Aipim Brasil, BRS Aramaris, BRS Caipira, BRS Dourada, BRS Formosa, BRS Gema de Ovo, BRS Kiriris, BRS Poti Branca, BRS Tapioqueira, BRS Verdinha, and IAC-90), and seven landrace varieties (Cascuda, Eucalipto, Fécula Branca, Irará, Mani Branca, Olho Junto, and Salangor).

The trials were arranged in a randomized complete block design, with six blocks and five plants of each genotype per block. The field was kept free of weeds by mechanical removal and crop management followed recommendations for cassava crops [28]. Harvesting was performed manually 10 months after planting for each of the two cultivation cycles.

Evaluation of root rot severity was performed via a visual assessment of the symptoms in the shoot portion of the plants by using the arbitrary rate scale described by Santiago et al. [29]:

0 no symptoms;
1 leaf chlorosis and/or wilting of the lower third;
2 leaf chlorosis and/or wilting of the middle third;
3 leaf chlorosis and/or wilting of the entire plant; and
4 complete defoliation and/or death of the plant.

A total of 14 evaluations were performed during each growing season (2014–2015 and 2016–2017). The first measurement was taken 15 days after planting (DAP), with seven subsequent evaluations made at weekly intervals. This was followed by four evaluations at 15-day intervals and three evaluations at 30-day intervals. The data obtained from the rate scale were converted into a general disease index (ω) (according to Czermainsk [30]) and used to calculate the area under the disease progress curve (AUDPC) (according to Campbell and Madden [31]).

The AUDPC was calculated using the following formula:

$$AUDPC=\sum [\left(\frac{y_{i}+y_{i+1}}{2})*(t_{i+1}-t_{i}\right)],$$

where *y*_*i*_ is the severity of the disease (based on the ω disease index) in the observed *i*; *y*_*i*+1_ is the severity of the disease at the time of the subsequent evaluation *i + 1*; *t*_*i*+1_ [20] is the time (days) of subsequent evaluation *i + 1*; and *t*_*i*_ is the time (days) at the time of observation *i*.

Agronomic traits were evaluated at the time of harvest. For this, all five plants in each plot were used and the following parameters were measured: (i) survival, expressed as the percentage of live plants; (ii) plant height, measured in meters; (iii) shoot yield, measured in t.ha^-1^; (iv) fresh root yield, measured in t.ha^-1^.

### Analysis of agronomic traits and root rot resistance

The analysis of phenotypic data was performed using linear mixed models. For this purpose, the best linear unbiased predictors (BLUPs) for each genotype were estimated by combining data from each of the two years using the following model: *y*_*ijk*_ = μ + *g*_*i*_ + *e*_*i*_ + (*ge*)_*ij*_ + *ε*_*ijk*_, where *y*_*ijk*_ is the response variable from the k^th^ repetition of the i^th^ genotype of the j^th^ year of evaluation; μ is the general average of the experiment; *g*_*i*_ is the random effect of the ith genotype; *e*_*i*_ is the fixed effect of the j^th^ year of evaluation; (*ge*)_*ij*_ is the effect of the interaction of the i^th^ genotype of the j^th^ year of evaluation; and *ε*_*ijk*_ is the experimental error associated with the ijk^th^ observation. The mixed model was analyzed using the lme4 package [32] from R software, version 4.1.1 [33].

Based on the BLUPs, Pearson’s correlation test was conducted with the help of the GGally package; the cluster analysis was conducted using the cluster, fpc, and mclust packages; the principal component analysis (PCA) was conducted using the factoextra, devtools, and FactoMineR packages; and the comparative distribution (boxplot) was conducted using ggpubr. All packages were implemented with R software, version 4.1.1 [33].

Data from the AUDPC underwent analysis of variance, and the means were grouped by Scott-Knott tests at the 5% significance level using the laercio package. The graphs were built using ggplot2, which was also implemented with R software, version 4.1.1 [33].

To select the genotypes with the best performance in the field infested by root rot, the values assigned to each characteristic were added to obtain the sum of the *ranks* that represents the classification of the genotypes, according to what was described by Mulamba and Mock [34]. This was conducted based on a hierarchization of the genotypes for each characteristic by assigning higher absolute values to those with better performance. To represent differences of importance for the breeding process, different weights normally used in the Embrapa’s cassava breeding pipeline were provided for the evaluated characteristics of each agronomic trait. The selection index was formulated as *IS* = (PH*1) + (ShY*3) + (FRY*5) + (ω* − 5) + (Su*5), in which *PH* is the plant height, *ShY* is the shoot yield, *FRY* is the fresh root yield, *ω* is the index of disease, and *Su* is clone survival. Since our main goal was root rot resistance and tuberous root yield, the highest weight was given to the agronomic traits associated with root yield and disease impact, followed by shoot yield, representing the total amount of aerial parts produced, and, less important for this breeding phase, the plant height. The expected gains from selection (GS) were obtained using the formula $GS={\hat{x}}_{sel-}{\hat{x}}_{pop}$, where ${\hat{x}}_{sel}$ is the average of the selected genotypes and ${\hat{x}}_{pop}$ is the average of all genotypes.

### DNA extraction and genotyping

DNA was extracted from young leaves according to the CTAB protocol [35]. The quality of the DNA was evaluated by quantification in 1% (w/v) agarose gel stained with ethidium bromide (1.0 mg.L^-1^) in TBE buffer 0.5x (45 mM Tris-borate, 1 mM EDTA, and q.s.p of distilled water), visualization in UV light, and registration using a Gel Logic 212 Pro photodocumenter (Carestream Molecular Imaging, New Haven, USA) through visual comparison with a series of known DNA concentrations of the lambda phage (Invitrogen, Carlsbad, CA). The DNA was diluted in TE buffer (Tris-HCl 10 mM and EDTA 1 mM) for a final concentration of 60 ng.Ml^-1^. The quality was verified by the digestion of 250 ng of the genomic DNA from 10 random samples with the restriction enzyme *EcoRI* (New England Biolabs, Boston, USA) at 65 °C for two hours, followed by visualization in agarose gel.

The DNA samples were genotyped using the basic genotyping-by-sequencing (GBS) protocol [36], by which the DNA was digested by the enzyme ApeKI [37] (a type II restriction endonuclease that recognizes a five-base degenerate sequence [GCWGC, where W is A or T]) with lengths of 100 bp. The connection between the ApeKI-cut fragments and the adapter was made after the digestion of the samples and the implementation of a multiplex system with 192 samples to perform sequencing. GBS was performed using a Genome Analyzer 2000 (Illumina, Inc., San Diego, CA).

Quality checks involved the removal of data missing more than 20% of loci, lower allelic frequency <0.01 per loci, and removal of individuals with more than 80% of data lost by chromosome. This identified a total of 27,045 biallelic single nucleotide polymorphisms (SNPs), with an allelic correlation of ≥0.8. The average coverage of SNPs was 1,503 markers per chromosome.

### Population structure and genomic association analysis

The association analysis of the data on resistance to root rot disease and productive attributes under root rot pathogen-infested field conditions was performed based on the general linear model (GLM), with the PCA a fixed effect as a covariable to explain the population structure. It was also performed based on the mixed linear model (MLM), with population structure (PCA) and kinship matrix included as covariables. Additionally, the analysis was performed based on a fixed and random model circulating probability unification (FarmCPU), with population structure (PCA) and kinship matrix as covariables. These models included additional algorithms to solve confusion problems between test and covariable markers. All models were implemented using the rMVP package [38].

To avoid the presence of false positives caused by the population structure, the first five principal components were adjusted as covariables in the model. The genomic kinship matrix of cassava genotypes was obtained according to van Raden [39], in which $\cup =\frac{ZZ^{\prime }}{2\sum \rho_{i}\;(1-\rho_{i})}$, where *Z M P* M are the matrix of markers, and P is the matrix of allelic frequencies expressed by 2(*ρ*_*i*_ − 0,5). This kinship matrix was combined with the population structure to control the occurrence of Type I errors. The limit for identifying significant associations between SNPs and phenotypes was estimated by the Bonferroni test (p<0.05).

Significant SNPs were found in the reference genome of cassava v6.1 [40], which was entered into the Phytozome v11.0 database by the JBrowse tool [41]. The functional annotations of all genes were identified by the *PhytoMine* tool of Phytozome v11.0.

## Results

Through morphological and molecular methods, 71 isolates were identified from 16 soil samples collected at different points in the experimental field. According to ITS rDNA sequencing of the isolates, a total of six genera were present in the experimental cassava field, with a 77% relative prevalence in terms of the isolation frequency of fungi in the genus *Fusarium* spp., 10% in *Dipodascus* sp., 8% in *Lasiodiplodia* spp., and 1% each in *Gongronella* sp., *Myrothecium* sp., and *Colletotrichum* sp.

Although *Gongronella* sp., *Myrothecium* sp., *Colletotrichum* sp., and *Dipodascus* sp. were recorded, these isolates did not elicit symptoms in detached cassava roots and were considered non-pathogenic. On the other hand, isolates from *Fusarium* spp. and *Lasiodiplodia* spp. species caused typical lesions on the cassava varieties (in vitro), thereby enabling their re-isolation and completion of Koch’s postulates.

The cassava genotypes exhibited significant differences in agronomic and resistance traits. In general, the percentage of survival (Su) per genotype ranged from 0.83% to 90.88%. The clones with the highest survival were BGM-1171 (90.88%), IAC90 (88.89%), and BGM-0878 (88.89%), and those with the lowest Su values were BGM-1027 (0.83%), BGM-2020 (0.91%), BGM-1464 (0.91%), BGM-1832 (0.91%), and BGM-1865 (0.91%).

The disease index (ω) varied between 7.80% and 100% severity. The genotypes with the lowest disease index showed the highest survival: IAC90 (7.80), BGM-1171 (11.37), and BGM-0878 (13.21). On the other hand, genotypes with the highest disease rate were BGM-2061 (100.00), BGM-1345 (100.00), and BGM-1440 (97.72).

Plant height (PH) varied from 1.30 m to 2.39 m, with the tallest genotypes BGM-0509 (2.39 m), BGM-0877 (2.37 m), and BGM-0945 (2.21 m), and the lower genotypes BGM-2066 (1.30 m), BGM-1482 (1.32 m), and BGM-0822 (1.39 m). Shoot yield trait (ShY) ranged from 0.30 t ha^-1^ to 27.03 t ha^-1^, and the genotypes BGM-0398 (27.03 t ha^-1^), BGM-1206 (21.16 t ha^-1^), and BGM-0209 (20.59 t ha^-1^) were the most promising. Genotypes BGM-2061 (0.30 t ha^-1^), BGM-0148 (0.42 t ha^-1^), and Irará (0.58 t ha^-1^) showed the lowest ShY. Finally, for fresh root yield (FRY), the amplitude of BLUPs ranged from 0.01 t ha^-1^ to 17.88 t ha^-1^, with genotypes BRS Kiriris (17.88 t ha^-1^), Olho Junto (15.64 t ha^-1^), and BGM-0624 (14.59 t ha^-1^) recording the highest FRY, and BGM-2061 (0.01 t ha^-1^), BGM-0190 (0.02 t ha^-1^), and BGM-1884 (0.02 t ha^-1^) recording the lowest FRY.

Due to high plant rate mortality, the genotypes BGM-0598, BGM-1027, BGM-1345, BGM-1365, BGM-1464, BGM-1832, BGM-1865, BGM-1867, and BGM-2020 had no values assigned to any traits.

**Fig 1:**
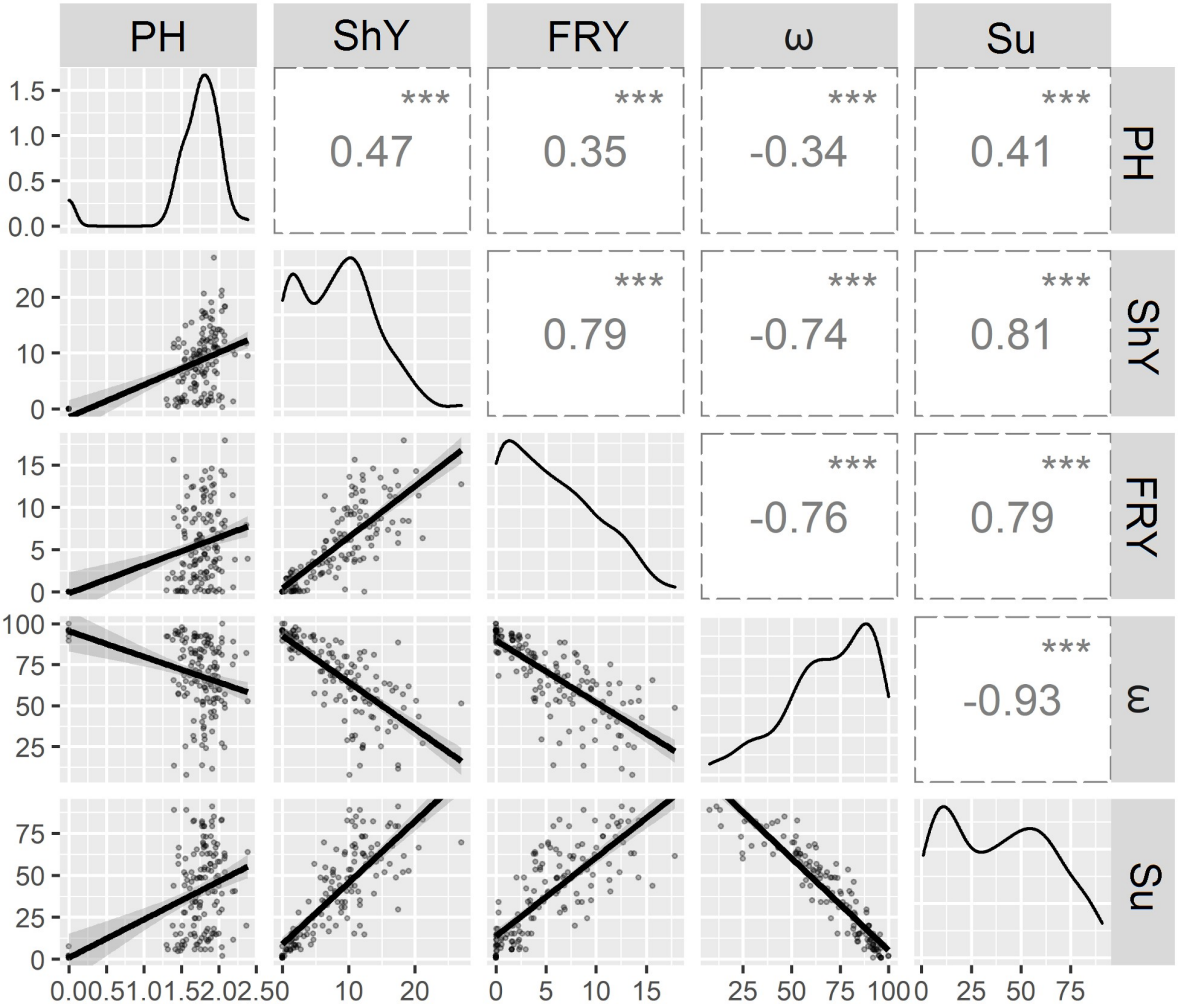
Pearson correlations with four agronomic traits derived from the evaluation of 148 cassava genotypes cultivated in areas infested by root rot pathogens. PH: plant height (m). ShY: shoot yield (t ha^-1^). FRY: fresh root yield (t ha^-1^). ω: disease index. Su: survival (%). Level of significance α: *** = 0.001; ** = 0.01.

In general, all correlation coefficients were significant (Fig 1). The disease index (ω) indicated a negative association with the other agronomic traits, with a correspondingly high negative correlation with the survival of plants in the field (-0.93, α = 0.001), which was also the case for fresh root yield and shoot yield (-0.76, α = 0.001 and -0.74, α = 0.001, respectively). A moderate negative correlation was also observed between ω and plant height (-0.34, α = 0.001). This result suggests that the severity of the disease directly affects the agronomic performance of most cassava genotypes and, consequently, the crop yield.

In contrast, there was a high and positive correlation between survival and characteristics such as shoot yield (0.81, α = 0.001) and fresh root yield (0.79, α = 0.001). Therefore, the survival of clones throughout the crop cycle is of fundamental importance when evaluating genetic resistance to root rot, considering their higher shoot and fresh root yields.

The correlations between plant height and other characteristics were of moderate magnitude (i.e., 0.41, α = 0.001 with plant survival; 0.35, α = 0.001 with fresh root yield; and 0.47, α = 0.001 with shoot yield). This indicates that taller plants do not necessarily have higher shoot or fresh root yields.

**Fig 2:**
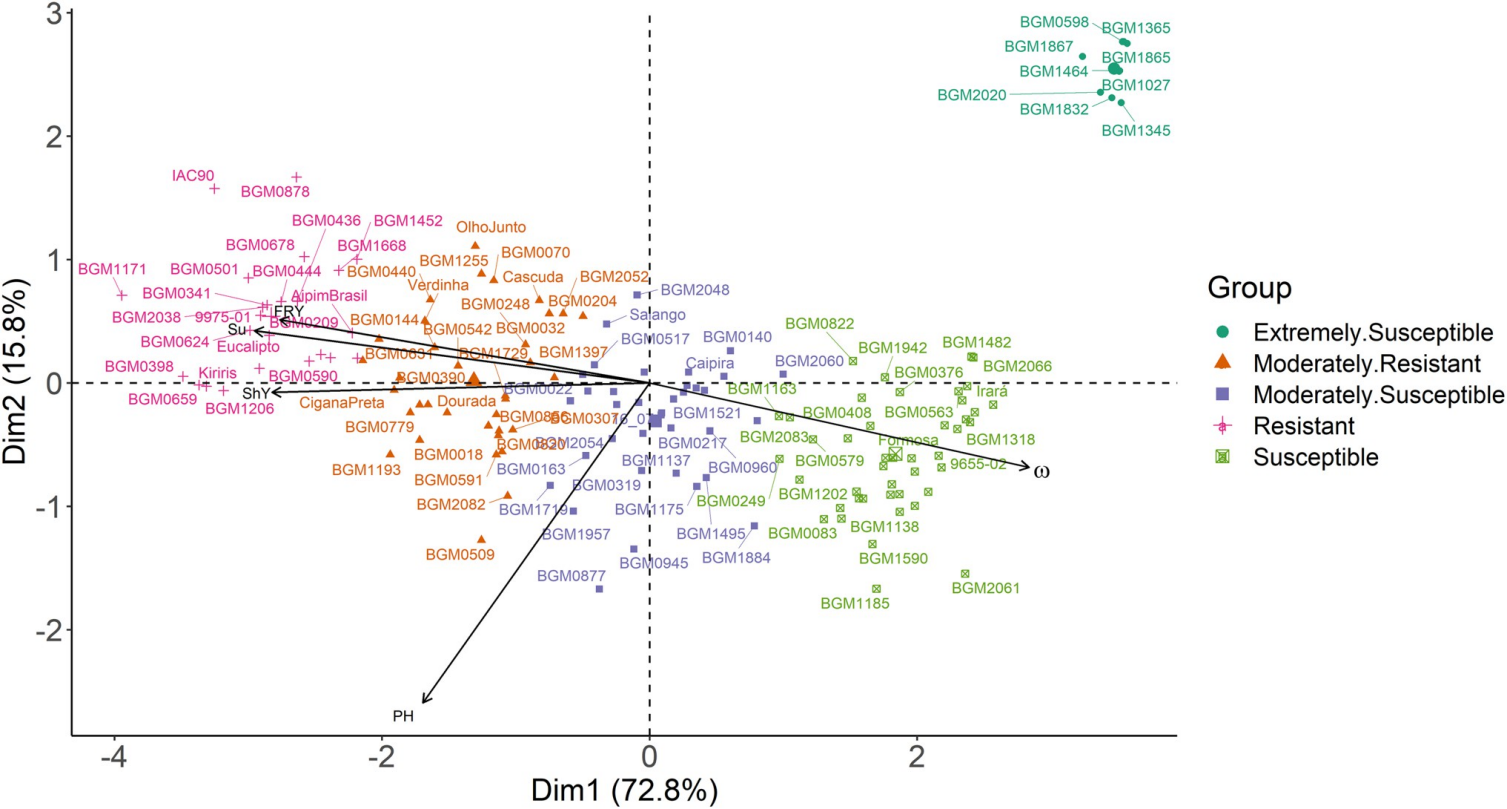
Principal components analysis for agronomic traits and root rot resistance in 148 cassava genotypes grown in an area naturally infested with root rot pathogens. PH: plant height (m). ShY: shoot yield (t ha^-1^). FRY: fresh root yield (t ha^-1^). ω: disease index. Su: survival (%).

The 148 genotypes were divided into five groups based on the PCA, with the number of genotypes per group 9 (G3), 24 (G4), 35 (G2), 36 (G1), and 44 (G5). The first two principal components captured 88.6% of the total variation associated with the data on plant height, fresh root and shoot yields, disease index, and survival (Fig 2), which indicates a good capacity to represent the genetic diversity of cassava genotypes in terms of resistance to root rot in field conditions. As per the Pearson correlation, analysis of the principal components verified that the disease index was negatively correlated with the other variables—mainly with survival. Likewise, certain characteristics (i.e., survival, shoot and fresh root yields) had strong positive correlations. However, although plant height was positively correlated with survival, as well as shoot and fresh root yields, it behaved as an independent variable in practice.

**Fig 3:**
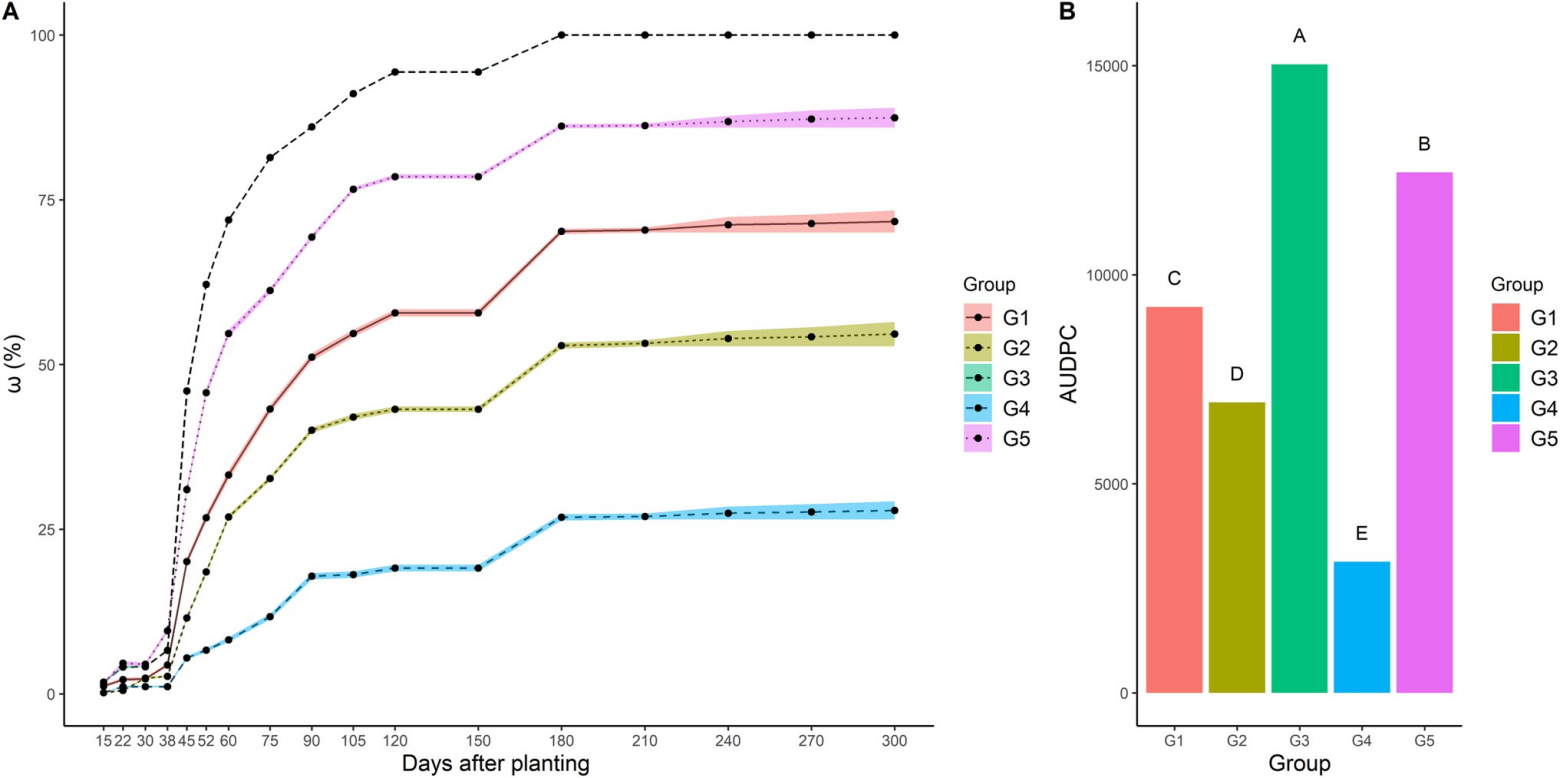
Mean values for the disease index (ω) and area under the disease progress curve (AUDPC) of the five groups formed via cluster analysis from the 148 cassava genotypes evaluated in areas infested by root rot pathogens between 15 and 210 days after planting. Means followed by the same letter belong to the same group based on the Scott-Knott test (p<0.05).

All groups were composed of genotypes that presented characteristic symptoms of the disease, such as the wilting and yellowing of leaves, and—in more extreme cases—genotypes that did not take root during an average period of 15 to 38 DAP (considered dead plants). It was observed that the greatest damage occurred after 45 DAP and that symptoms in the plant shoots showed a tendency to stabilize after 180 DAP. Groups G3 and G5 presented the highest rates of disease in all evaluations, followed by G1, G2, and G4 (Fig 3A).

The AUDPC mean comparison showed that there was variation in disease progression, with a significant difference between the five groups evaluated for root rot resistance under field conditions (Fig 3B). Therefore, these groups were classified according to level of root rot resistance based on the severity of symptoms. Groups G3 and G5 presented the highest AUDPC values, indicating faster progression of the disease during the evaluation period; therefore, these groups were classified as extremely susceptible (ES) and susceptible (S), respectively. Group G1 was classified as moderately susceptible (MS), G2 as moderately resistant (MR), and G4, with the lowest AUDPC value, was classified as resistant (R).

### Classification of genotypes by resistance to root rot

Genotypes belonging to the group considered extremely susceptible (G3), with nine genotypes (S3 Table), had the lowest survival values (1.97%), the highest disease index values (ω average = 95.11%), and was noticeably lacking in the evaluation of agronomic attributes (Table 1). Next, the group susceptible (G5), with 44 genotypes (S4 Table), presented the second lowest survival value (12.63%), as well as the second highest average values, for disease index (89.70%), plant height (1.70 m), shoot yield (2.51 t ha^-1^), and fresh root yield (1.25 t ha^-1^) compared to the other groups, thereby indicating its high susceptibility to root rot. Among these genotypes are clones and improved varieties (9655–02, BRS Formosa, and BRS Poti Branca), as well as landraces (Fécula Branca, Irará, and Mani Branca).

**Table 1:** Averages for survival, disease index, plant height, and shoot and fresh root yields for the groups formed by cluster analysis, with the expected selection gain (GS%) relative to the original population.

**Group**	**Survival (%)**			
	Minimal	Maximal	Mean	GS%
M. Susceptible—G1	14.74	56.65	37.66	-3.21
M. Resistant—G2	35.16	73.24	56.74	45.85
E. Susceptible—G3	0.83	7.56	1.97	-94.94
Resistant—G4	59.87	90.88	**76.80**	**97.40**
Susceptible—G5	1.75	34.21	12.63	-67.55
Original Population	0.83	90.88	38.90	
**Group**	**Disease Index (%)**			
	Minimal	Maximal	Mean	GS%
M. Susceptible—G1	57.47	88.57	73.87	6.44
M. Resistant—G2	29.47	75.17	57.57	-17.05
E. Susceptible—G3	89.59	100.00	95.11	37.03
Resistant—G4	7.80	52.88	**33.13**	**-52.27**
Susceptible—G5	78.94	100.00	89.70	29.24
Original Population	7.80	100.00	69.41	
**Group**	**Plant Height (m)**			
	Minimal	Maximal	Mean	GS%
M. Susceptible—G1	1.46	2.37	1.79	7.06
M. Resistant—G2	1.40	2.39	**1.83**	**9.53**
E. Susceptible—G3	0.00	0.00	0.00	-
Resistant—G4	1.40	2.08	**1.83**	**9.41**
Susceptible—G5	1.30	2.19	1.70	1.97
Original Population	0.00	2.39	1.67	
**Group**	**Shoot yield (t ha** ^**-1**^ **)**			
	Minimal	Maximal	Mean	GS%
M. Susceptible—G1	4.82	17.44	8.79	7.33
M. Resistant—G2	6.30	19.03	12.08	47.55
E. Susceptible—G3	0.00	0.00	0.00	-
Resistant—G4	9.79	27.03	**15.08**	**84.24**
Susceptible—G5	0.30	8.07	2.51	-69.32
Original Population	0.00	27.03	8.19	
**Group**	**Fresh root yield (t ha** ^**-1**^ **)**			
	Minimal	Maximal	Mean	GS%
M. Susceptible—G1	0.02	9.11	4.97	-7.38
M. Resistant—G2	3.78	15.64	8.16	51.90
E. Susceptible—G3	0.00	0.00	0.00	-
Resistant—G4	5.75	17.88	**11.47**	**113.53**
Susceptible—G5	0.01	3.56	1.25	-76.72
Original Population	0.00	17.88	5.37	

The group considered moderately susceptible (G1) was composed of 36 genotypes (S5 Table). The genotypes belonging to this group presented intermediate values for plant height (1.79 m), shoot yield (approximately 8.8 t ha^-1^), and fresh root yield (around 5.0 t ha^-1^). However, the same group also presented the third highest rate of disease (73.87%), as well as survival below 40%. These genotypes include improved clones and varieties (*BRS Caipira*, *BRS Gema de Ovo*, *BRS Tapioqueira*, *2002-01-01*, and *2002-16-07*), as well as the landrace *Salangor*.

In general, the genotypes belonging to these three groups presented characteristic symptoms of the disease at an early stage of plant development, and the infection resulted in death during the emergence of shoots or up to 40 DAP. Therefore, none of the clones or varieties belonging to these groups are identified for planting in areas with a history of the disease.

The group moderately resistant (G2), formed by 35 genotypes (S6 Table), presented the highest average plant height (1.83 m), second highest survival rate (56.74%), an average shoot yield of 12.0 t ha^-1^, an average fresh root yield of 8.0 t ha^-1^, and a moderate disease index ($\bar{\omega }=57.57$). This group includes the landraces *Cascuda*, *BRS Aramaris* (sin = Cigana Preta), and *Olho Junto*, as well as the improved varieties *BRS Dourada* and *BRS Verdinha*.

The 24 genotypes in the resistant group (G4) (S7 Table) presented the highest average plant height (1.83 m) (no different from the MR group for this characteristic), as well as the highest average survival rate (76.80%), the lowest disease index ($\bar{\omega }=33.13$), and the highest averages for shoot yield (15.0 t ha^-1^) and fresh root yield (11.5 t ha^-1^). This group contained the improved varieties *BRS Aipim Brasil*, *IAC-90*, and *BRS Kiriris*, the hybrid *9975–01*, and the landrace *Eucalipto*. Despite the existence of promising genotypes for agronomic characteristics of interest, no genotype has been completely immune to root rot.

In addition to showing the highest resistance to root rot in a field with high inoculum potential, the genotypes within the resistant group also showed the best agronomic performance. Therefore, to assist the choice of parental genotypes for the development of segregation progeny in the cassava breeding program, a selection was made based on the sum of the rankings for each genotype and each characteristic evaluated, which was based on the phenotypic BLUPs of the 10 best genotypes (Top 10) from among the 24 that comprised this group (Table 2).

**Table 2:** Averages for disease index (ω), survival (Su), plant height (PH), shoot yield (ShY), and fresh root yield (FRY) considering only the 10 best genotypes according to the selection index and expected selection gain (GS%) relative to the original population.

Top10		Parameters									
Sum. Rk (MM)	Genotypes	ω (%)	GS (%)	Su (%)	GS (%)	PH (m)	GS (%)	ShY (t ha^-1^)	GS (%)	FRY (t ha^-1^)	GS (%)
1	BGM1171	11.37	**-83.62**	90.88	**133.60**	1.91	14.68	17.49	113.61	12.56	133.92
1	BRS Kiriris	48.65	-29.90	61.48	58.03	2.08	**24.60**	18.31	123.74	17.88	**232.89**
1	BGM0659	52.88	-23.81	82.44	111.90	2.04	21.97	20.22	146.99	14.26	165.56
2	Eucalipto	24.62	-64.54	63.10	62.19	2.08	24.49	12.08	47.58	13.81	157.08
3	BGM0398	51.34	-26.03	69.55	78.76	1.93	15.78	27.03	**230.17**	12.71	136.70
4	BGM1206	27.05	-61.03	83.04	113.45	2.04	22.12	21.16	158.51	6.36	18.40
5	BGM0624	42.82	-38.30	82.06	110.93	1.91	14.16	13.71	67.50	14.59	171.76
6	BGM2169	52.10	-24.94	63.10	62.19	1.88	12.88	14.82	81.07	13.50	151.41
7	BGM1190	46.40	-33.15	85.00	118.49	1.93	15.84	11.10	35.58	11.67	117.26
8	BGM0209	43.16	-37.82	66.32	70.47	1.80	8.00	20.59	151.59	11.38	111.91
	Minimal	11.37		61.48		1.80		11.10		6.36	
	Maximal	52.88		90.88		2.08		27.03		17.88	
	Mean	40.04	**42.31**	74.70	**92.00**	1.96	**17.45**	17.65	**115.63**	12.87	**139.69**
Original Population		69.41		38.90		1.67		8.19		5.37	

Compared to the original population, there was a high average genetic gain with the selection of the 10 best genotypes for most of the characteristics evaluated (Table 2). The expected gains from the selection of the Top 10 were 115.63% for shoot yield, 139.69% for fresh root yield, 17.45% for plant height, 92% for plant survival in the field, and a root rot severity reduction of 42.31%. Within the Top 10, the genotype BGM-1171 presented the lowest rate of disease and highest rate of survival in the infested field. The variety BRS Kiriris produced the highest fresh root yield, followed by the landrace Eucalipto, which produced the highest plant height. The genotype BGM-0398 produced the highest shoot yield. The results indicate that these genotypes are potential parentals to be recombined and used for the next stages of the breeding program.

**Fig 4:**
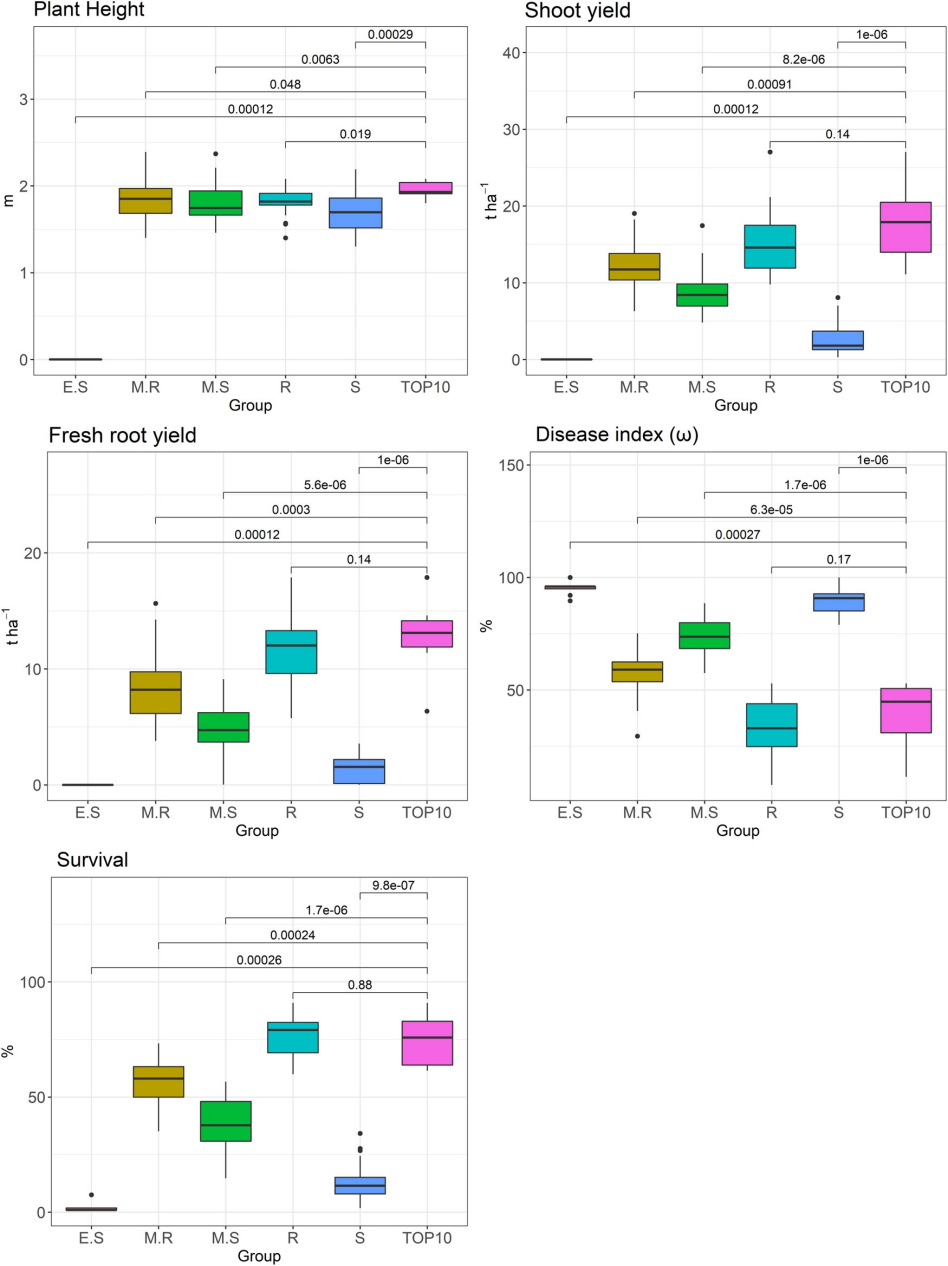
Boxplot of the clusters formed based on plant height, shoot and fresh root yields, survival, and disease index (ω) evaluated for 148 cassava genotypes planted in a field naturally infested with root rot pathogens. ES: extremely susceptible; S: susceptible; MS: moderately susceptible; MR: moderately resistant; R: resistant.

The means were compared by using a t-test on the five groups of the cluster analysis and the Top 10 group, as well as on the distribution of the numerical variables among the six groups (Fig 4). Regarding plant height, the mean of the Top 10 group was significantly different from those of the other groups. However, for shoot yield, fresh root yield, disease index, and survival, there was no statistical difference for the means of the resistant group. Therefore, the mean values of the genotypes belonging to these groups were higher than those of the remaining genotypes for all agronomic characters of interest. However, when comparing the distribution of characteristics between the Top 10 and the resistant group, it was found that for the shoot and fresh root yields, the Top 10 had a higher median than the resistant group. Additionally, the Top 10 also showed a lower dispersion between the quartiles for fresh root yield. However, the resistant group had a lower median for the disease index (ω), which indicates that the genotypes generally had lower disease severity scores. Regarding survival in the field, the resistant group presented a lower dispersion in the quartiles and a slightly higher median.

**Fig 5:**
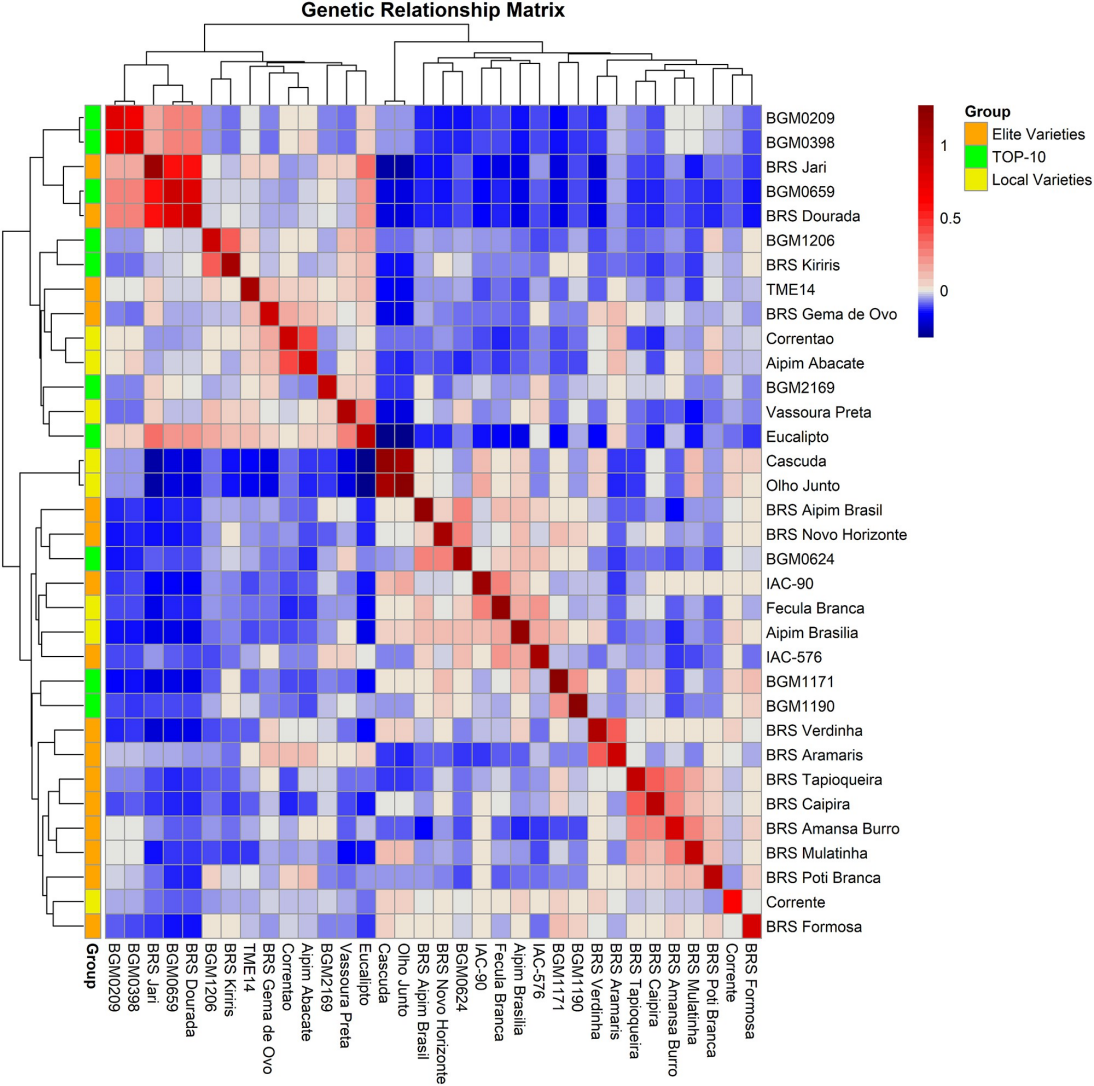
Heatmap of the genomic kinship of 10 putative resistance sources to cassava root rot disease, 16 elite varieties, and *7* cassava landraces based on analysis of 72,023 single nucleotide polymorphisms markers.

Based on the SNP markers from the pairwise comparison of cassava genotypes belonging to the Top 10 group, the estimates of genomic kinship varied from—0.183 to 0.671, with an average of—0.00046 (Fig 5). The negative values of genomic kinship indicate pairs of individuals sharing few alleles compared to what is expected based on their allelic frequencies. The highest estimates of observed genomic kinship were between the genotypes BGM-0209 × BGM-0398 (0.671), BGM-1206 × BRS Kiriris (0.346), BGM-0659 × BGM-0209 (0.282), and BGM-0659 × BGM-0398 (0.277), as well as between BGM-1171 × BGM-1190 (0.197). Considering the need to maximize the potential for selection gains, crossover between these genotypes with high degrees of genomic kinship should be avoided.

On the other hand, the most highly contrasted genotypic combinations within the Top 10 group were among the following genotype pairs: BGM-1171 × BGM-0659 (-0.183), BGM-1171 × Eucalipto (-0,168), BGM-1171 × BGM-0209 (-0.160), BGM1171 × BGM-0398 (-0.155), BGM-0624 ×BGM-0209 (-0.147), BGM-1190 × BGM-0398 (-0.119), BGM-1190 × BGM-0209 (-0.115), and BGM-1190 × BGM-0659 (-0.106). The genotypes BGM-1171 and BGM-1190 exhibited the lowest degrees of kinship with the other selected resistance sources and are, thus, considered potential parental genotypes aimed at increasing root rot resistance.

In addition to the Top 10 group, the genomic kinship matrix also included 16 elite varieties and 7 landraces commonly used as parentals with high genetic value for root and starch yields. In this case, the focus would be to select elite cassava parentals with minimal genetic kinship to the sources of root rot resistance, thereby maximizing the chances of obtaining segregation clones. As a result, putative resistance sources BGM-0209, BGM-0398, and BGM-0659 showed negative kinship values with 14 out of the 16 elite varieties of cassava, with the highest contrasting genotypic combinations for BGM-0659 involving the varieties BRS Verdinha (-0.184) and IAC-90 (-0.164). For BGM-0209, the smallest degree of kinship was observed with the varieties BRS Novo Horizonte (-0.146) and BRS Verdinha (-0.135). Finally, the smallest genomic kinships with genotype BGM-0398 were observed with the varieties BRS Novo Horizonte (-0.142) and BRS Aipim Brasil (-0.128).

Other putative sources of root rot resistance—such as BGM-0624, BGM-1206, BGM-1190, and BRS Kiriris—showed negative values for kinship estimations with 13 other elite varieties. For BRS Kiriris, the elite varieties with the lowest degree of genomic kinship were BRS Caipira (-0.126) and BRS Mulatinha (-0.119). For genotype BGM-0624, the lowest degrees of genomic kinship were observed with BRS Aramaris (-0.123) and BRS Amansa Burro (-0.114). For resistance source BGM-1206, the highest contrasting elite varieties were BRS Verdinha (-0.114) and IAC-576 (-0.114). For BGM-1190, the highest contrasting elite varieties were BRS Dourada (-0.109) and BRS Jari (-0.105).

On the other hand, the Eucalipto genotype had a low degree of kinship with 11 elite varieties, with the highest contrasting BRS Verdinha (-0.157) and BRS Mulatinha (-0.155). BGM-1171 also presented low kinship estimates with 10 elite varieties; the two highest contrasting genotypic combinations were BRS Jari (-0.197) and BRS Dourada (-0.183). Finally, the source of resistance to root rot, BGM-2169, had a low degree of kinship with six elite varieties, especially with BRS Novo Horizonte (-0.094) and BRS Poti Branca (-0.069).

When compared to the landraces (i.e., Correntão, Aipim Abacate, Vassoura Preta, Cascuda, Olho Junto, Fécula Branca, and Corrente), genotype BGM-0659 showed negative values of kinship with all varieties, with the highest contrasting combinations observed with Cascuda (-0.196) and Olho Junto (-0.195). The resistant genotypes BGM-2169 and BGM-1190 showed low genomic kinship with six cassava landraces, with potential crosses between BGM-2169 × Cascuda (-0.127) and Olho Junto (-0.125), and between BGM-1190 × Aipim Abacate (-0.106) and Correntão (-0.083) showing the least kinship.

The sources of resistance to root rot (i.e., BGM-0209, BGM-0398, BGM-0624, and BRS Kiriris) presented low kinship with only five landraces, with the highest contrasting combinations as follows: BRS Kiriris × Cascuda (-0.147) and Olho Junto (-0.146); BGM-0624 × Aipim Abacate (-0.133) and Correntão (-0.086); BGM-0209 × Fécula Branca (-0.113) and Vassoura Preta (-0.083); and BGM-0398 × Fécula Branca (-0.112) and Cascuda (-0.084). However, for BGM-1171, Eucalipto, and BGM-1206, the crosses with the greatest potential for generating segregated populations for resistance were between BGM-1171 × Correntão (-0.113) and Cascuda (-0.106); Eucalipto × Olho Junto (-0.325) and Cascuda (-0.316); BGM-1206 × Cascuda (-0.085) and Olho Junto (-0.079).

### Association mapping

**Fig 6:**
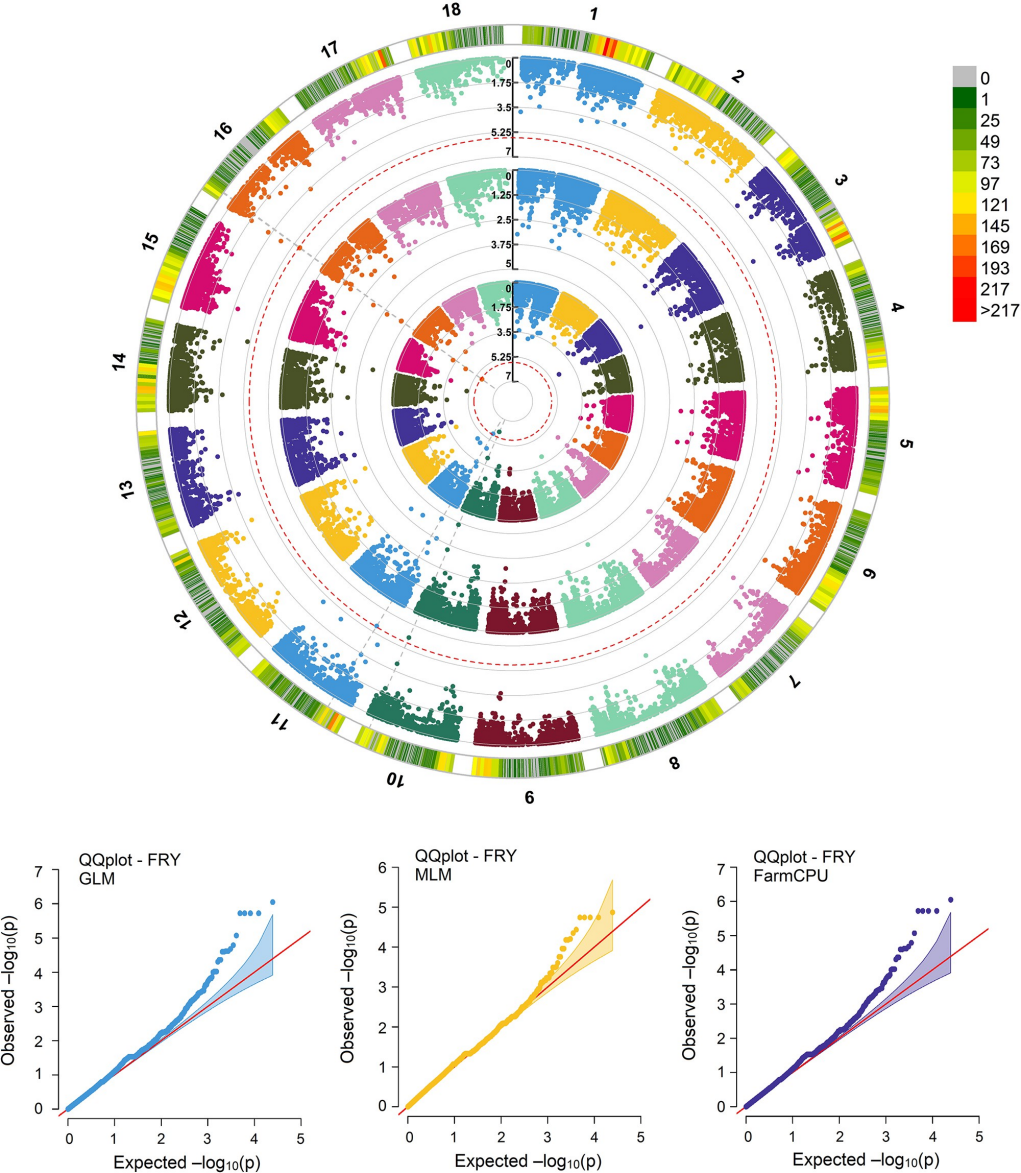
Circular Manhattan plot indicating single nucleotide polymorphisms associated with fresh root yield according to the FarmCPU (outer circle), MLM (middle circle), and GLM (inner circle) models. The dotted red line indicates the threshold for significant association, after applying the Bonferroni correction (p<0.05). The quantile-quantile (QQ) plots are of expected versus observed significance levels for each GWAS model. The red diagonal lines indicate the null hypothesis, where the observed and expected P-values would be situated if there were no associations. The shaded area indicates a 95% confidence interval.

Based on the association mapping of 27,045 SNPs distributed across 18 cassava chromosomes, markers significantly associated with the characteristic yield of roots were identified by the FarmCPU and GLM models after applying the Bonferroni correction (p<0.05) (Fig 6). The genomic kinship matrix effectively represented the population structure in the different GWAS models, since no other associations were observed other than expected low levels of significance on the quantile-quantile (QQ) plot (Fig 6). For the other evaluated traits, such as plant height, shoot yield, disease index, and survival, no markers were identified as being in linkage disequilibrium.

The five major SNPs associated with cassava fresh root yield were in different genomic regions located on chromosomes 10, 11, and 16 (Table 3). The SNPs on chromosomes 11 and 16 explained the same proportion of phenotypic variance (0.1655), while S10_24373837 explained a little more of this variance (0.1732).

**Table 3:** Relationship of single nucleotide polymorphisms markers and traits after applying the Bonferroni correction (p<0.05) for fresh root yield in cassava genotypes.

SNP	Chromosome	p-value (-log10)	r2	Transcript	Description	Function
S10_24373837	10	60.485	0.1732	Manes.10G131800.1	lung seven transmembrane receptor-like protein	Transmembrane signal receiver
S11_5550733	11	57.228	0.1655	Manes.11G056800.1	post-illumination chlorophyll fluorescence increase	Response to photoinhibition conditions
S11_5585466	11	57.228	0.1655	Manes.11G057200.1	leucine-rich repeat-containing protein	Proteína de resposta antimicrobiana
S16_2375953	16	57.228	0.1655	Manes.16G024300.1	u3 small nucleolar rna-associated protein 18 (utp18)	WD repeat protein
S16_2624456	16	57.228	0.1655	Manes.16G026800.1	e3 ubiquitin-protein ligase rnf5	Ubiquitination by the enzyme E3 ligase

SNP S10_24373837 is located on chromosome 10 in the *Manes*.*10G131800*.*1* gene, which is described as a lung seven transmembrane receptor-like protein, a transmembrane signal receiver that transmits physiological signals from outside into the cell (GPCRs) via G proteins. GPCRs constitute the largest known superfamily of transmembrane-like receptors that respond to a wide variety of extracellular stimuli, including peptides, lipids, amino acids, hormones, and environmental stimuli [42].

The significant SNPs S11_5550733 and S11_5585466, located on chromosome 11, are related to the genes *Manes*.*11G056800*.*1* and *Manes*.*11G057200*.*1*, respectively. The transcript for *Manes*.*11G056800*.*1* is described as post-illumination chlorophyll fluorescence increase protein, which encodes a chloroplastidial protein that is an essential component for non-photochemical reduction mediated by the NADH dehydrogenase of the plastoquinone pool during the transport of chlororespiratory electrons. Therefore, it is involved in cellular acclimatization to heat, chlororespiration processes, and non-photochemical elimination [42]. *Manes*.*11G057200*.*1* (a leucine-rich repeat-containing protein) is an evolutionary domain preserved in many proteins associated with innate immunity in plants, serving as the first line of defense against pathogens [43].

On chromosome 16, SNP S16_2375953 is related to the *Manes*.*16G024300*.*1* gene (U3 small nucleolar RNA-associated protein 18-UTP18), which is involved in the nucleolar processing of pre-18S ribosomal RNA and ribosome assembly, while SNP S16_ 2624456 of *Manes*.*16 geneG026800*.*1* (E3 ubiquitin-protein ligase RNF5) is associated with the ubiquitination process of the target protein via the enzyme E3 ligase [44].

## Discussion

### Root rot resistance and production parameters

All genotypes, including highest survival rate and highest yield, had some drawbacks caused by the dry root rot (DRR) and black root rot (BRR) pathogens. Therefore, we showed the absence of full resistance and the high environment effect on the incidence and severity of disease for the genotypes evaluated. These findings agree with previous results, based on in vitro screening for resistance to DRR and BRR, where resistance to both diseases displayed quantitative behavior and was putatively controlled by minor genes with high environment effect [10, 20, 29].

Approximately 60% of the evaluated genotypes (G1, G3, and G5) were considered susceptible to root rot disease under field conditions. In cases of extreme susceptibility, there is no rooting or the plants exhibit early symptoms of disease during bud emergence and, consequently, die prematurely in the field. In general, disease severity evolved alongside the development of the tuberous roots, while the highest leaf symptoms occurred from 45 DAP, when the plants were beginning their growth and shoot formation processes. However, from 180 DAP, there was a tendency for the above ground symptoms to stabilize, such as wilting and even death of the plants. These observations indicate that the crucial period of plant-pathogen interaction (e.g., biochemical, physiological, and molecular responses to prevent the death of infected plants) should occur in the initial months of plant development.

On the other hand, the genotypes from groups G2 and G4, considered moderately resistant and resistant, respectively, had the highest averages for plant height and shoot yield. In this particular screening with root rot resistance sources, the main traits under selection were plant survival and agronomic performance in terms of shoot and fresh root yields. Although starch synthesis occurs in the leaves and is later transported to the reserve organ (i.e., the tuberous roots) [45], we observed that the genotypes with higher plant height and shoot yield were not necessarily the same as those that had higher fresh root yield in soils infested with cassava root rot pathogens. Since the mechanisms of root rot resistance are different in each plant tissue [29, 46], both groups likely present structural and biochemical mechanisms that may delay or block the entry and subsequent activity of pathogens in the stem.

Although the resistant (G4) group was composed of genotypes that—besides presenting a high level of performance for the production of plant biomass—had the lowest value of AUDPC, higher fresh root yield, lower disease rate, and higher survival in the field. Based on this finding, it is possible to hypothesize that the efficient capture and mobilization of nutrients for growth, as well as the ability to deal with external stressors, are key factors that contribute to a large cassava harvest [47].

The ranking of cassava genotypes facilitated the selection of 10 promising sources of resistance for simultaneous gains in plant survival and agronomic performance (in terms of shoot and fresh root yields), which are required to ensure production in areas infested with root rot pathogens. Since the average fresh root yield of Northeast Brazil is approximately 9.40 t ha^-1^ [48]—and because this data also includes locations without high inoculum potential—the averages of the Top 10 group highlight the potential of these genotypes in terms of yield in infested areas (11.10 to 27.03 t ha^-1^) and reinforce the prospect of considerable gains if they are used as parentals in the formation of segregated populations.

The only commercial variety belonging to the Top 10 group was BRS Kiriris, which has already been selected in Northeastern Brazil as a source of resistance to root rot [24] and performed the best in relation to plant height and fresh root yield. Notably, Eucalipto, a sweet landrace used for cooking, attained low disease indexes. These are genotypes with resistance to rotting that also have the advantage of already being adapted with favorable agronomic characteristics for cooking and industrial use. In contrast, eight accessions belonging to the BAG-Mandioca of Embrapa Mandioca e Fruticultura were described for the first time as possible sources of resistance to root rot. For example, BGM-1171 was the genotype with the best performance in terms of low disease rates and survival in the infested field. Additionally, BGM-0398 presented the highest average for shoot yield.

### Potential hybrid combinations for incorporating rot resistance into varieties with high genetic value

The selection of resistance sources is a fundamental step toward effective management of root rot. However, in addition to the availability of resistance sources, it is fundamental to understand the complexity of resistance inheritance and to have an efficient improvement process in which tests for the selection of disease resistance are integrated with other important crop agronomic characters for breeding programs to be successful [49].

The generation and evaluation of segregated populations from crosses between sources of resistance with elite varieties and landraces facilitates greater advances in the development of cassava varieties, the integration of resistance into the agronomic characteristics of interest, and adaptation to the main cultivation regions [50]. The data from tracking genetically segregated populations of crosses between resistant genotypes and elite varieties/landraces have been used to map quantitative trait loci (QTLs) that can be associated with resistance to some diseases affecting cassava, such as bacterial blight [51], cassava mosaic disease complex (CMD), and cassava brown streak disease (CBD) [52, 53]. However, no similar studies related to root rot disease have been conducted to date.

The GWAS of the 148 genotypes evaluated during two different cultivation cycles in areas with a natural occurrence of root rot disease detected five significant SNPs associated with fresh root yield. However, it did not allow identification of the genomic regions associated with the survival and disease index (ω) of cassava root rot under field conditions, despite the existence of important phenotypic variation in levels of resistance to root rot. The absence of SNPs significantly correlated with these two characteristics may be associated with the presence of minor genes that are unique to certain genetic backgrounds, which can, therefore, only be detected when a large panel of genotypes is analyzed. Some authors have demonstrated that increasing the size of the analyzed population has a greater impact on the detection power of the QTLs associated with a certain characteristic, since they allow the exploration of QTLs with minor effects [54].

In general, mapping resolution can be dramatically improved by increasing the number of genotypes with new recombination. Therefore, the next steps for this area of study will involve crossing root rot resistance sources and elite cassava varieties, as well as phenotyping segregated populations in areas with high disease infestation. Since thousands of individuals are usually generated in the breeding populations, this panel of genotypes, along with additional germplasm (improved varieties and landraces), will be used for further GWAS analysis in search of genes directly linked to survival and ω in field conditions.

Some previous reports have shown that resistance to cassava root rot has a polygenic character [23, 55]. Quantitative resistance is controlled by multiple minor genes on the plant, thereby promoting partial resistance to the pathogen and a decrease in the severity of symptoms and/or the progression of epidemics over time [56]. The genetic architecture of quantitative disease resistance has often been associated with a small number of detected QTLs and the effects of some major genes, alongside other genes with minor effects [57]. This reinforces the need for a future approach based on the study of segregation populations, resulting from the aforementioned targeted crossings to produce large populations that facilitate the detection of small effects locos associated with cassava root rot.

Additionally, upon understanding the mechanisms that enable a higher yield of cassava root in an area infested with root rot pathogens, it was determined that all SNPs significantly associated with fresh root yield under these conditions were located in gene regions associated with the aforementioned proteinaceous functions that act on defense mechanisms against biotic and abiotic stresses. Identification of the genes underlying the QTLs involved in resistance—or at least the molecular markers linked to them—enables the projection of marker-assisted approaches in the selection of resistant genotypes [58].

For example, the transcript *Manes*.*10G131800*.*1* encodes the receptor protein of the transmembrane signal coupled to the G protein (lung seven transmembrane receptor-like protein). The G-coupled receptors are the largest family of membrane proteins and act as key components of the signal transduction pathways in several plant species through different physiological processes [59, 60]. Although its mechanism of action has not yet been elucidated, the lung seven transmembrane receptor-like protein has been described in studies focused on the plant-pathogen interaction of mustard [61], the identification of QTLs, and candidate genes associated with resistance to the bacterial staining of beans [62] and host plants of *Candidatus Phytoplasma* [63].

*Manes*.*11G056800*.*1* is the coder for post-illumination chlorophyll fluorescence increase protein, which is associated with the response to photoinhibition under water deficit conditions [64]. Although they primarily affect the roots of cassava [17] and influence water absorption, the fungi associated with root rot can colonize the plant stems [29], thereby blocking the flow of sap and causing withering in the shoot, yield, and even the death of the plant. Thus, proteins that repair damage caused to the photosynthetic system of cassava due to various types of stress can help with plant growth and the development of its root system.

*Manes*.*11G057200*.*1* encodes the leucine-rich repeat-containing protein (NLR) that is involved in antimicrobial response. The resistance genes and encoding proteins of the NLR family (containing the leucine-rich nucleotide-binding domain) recognize the presence of pathogen effector proteins and elicit an immune response often associated with cell death at infection sites [65]. The NLR acts as a crucial regulator in the immune response of plants against various diseases, from those that cause vascular wilt and root/soil rot (e.g., *Phytophthora soya* [66] and *Fusarium virguliforme* [67], respectively) to diseases affecting the shoot yield of plants, such as wheat rust [68] and rubber tree *Corynespora* stain [69].

*Manes*.*16G024300*.*1* encodes WDR (U3 small nucleolar RNA-associated protein 18-UTP18), a diverse superfamily of regulatory proteins. Proteins containing WDR are involved in fundamental mechanisms such as signal transduction, chromatin modification, and transcription regulation [70, 71]. In plants, WDR has played important roles in activities such as floral development [72], flavonoid biosynthesis in tuberous roots, abiotic stress response [73], and the immune signaling and transcriptional regulation of chemical defenses [74–76].

Finally, *Manes*.*16G026800*.*1* (E3 ubiquitin-protein ligase RNF5) is associated with the ubiquitination process of the target protein by means of the enzyme E3 ligase. Ubiquitination is one of the most abundant types of post-protein modification (PTM) in plant cells and regulates a multitude of cell functions, from growth and development to responses to biotic and abiotic stimuli [77–80]. The enzymes E3 ubiquitin (Ub)-ligase are the most studied components of the ubiquitin cascade [78] and are involved in various aspects of plant immunity, ranging from pathogen perception to signal transduction and immune responses [80–85].

Based on the analysis of detached and artificially inoculated roots, the five SNPs associated with fresh root yield in the field did not coincide with the 38 significant SNPs associated with root rot resistance of detached roots [23]. Complex field interactions [86, 87] may not be reflected in studies with artificial inoculation in controlled environments [29, 58]. Thus, for future studies, besides a greater phenotypic correlation between genotypes, analyses that integrate different field conditions and several controlled conditions tests would make it possible to detect consistent QTLs in different environments, thereby considerably advancing progress in identifying the genomic regions involved in resistance against root pathogens [88–90].

Due to the devastating character of root rot, the need to develop cassava varieties with durable resistance to multiple pathogens is increasingly important for food security and economic development [23, 91]. However, since resistance is controlled by many genes, any method adopted must consider elements that make genetic improvement difficult (e.g., low heritability and high environmental influence), especially when no larger genes are present. Once the sources of resistance are identified, it is essential to establish strategies that allow the gradual accumulation of alleles favorable to the various genes that control resistance to root rot.

Overall understanding of the genetic mechanisms responsible for the various levels of resistance to root rot disease in field conditions among cassava genotypes remains underexplored. The studies developed to date have focused on searching for sources of resistance [10, 20] and identifying the possible genes involved in defense mechanisms [23, 55] using the in vitro methodology of root inoculation. However, in order to characterize the disease and the behavior of cassava genotypes in the field, it is necessary to consider the different defense mechanisms not only of the root system tissues, but also the shoot yields of the genotypes used in planting [29, 46]. Although the genes associated with plant response to pathogens and oxidative processes have been described as possibly involved in root resistance [23, 55], it is essential to identify and quantify which genes have constitutive expression, as well as which are subject to the control of temporal-specific and tissue-specific expression in cassava versus root rot interaction.

However, any new source of disease resistance must be selected within a genetic context that meets current requirements for yield and agronomic characteristics of interest [25]. Thus, recombination of the genotypes selected here should make it possible to obtain segregated populations with high average resistance levels, sufficient genetic variance for agronomic traits that meet end-user’s demand. In parallel, the mapping of QTLs related to yield in an infested area will enable the projection of marker-assisted approaches for the selection of resistant genotypes.

## Conclusion

Field observations indicated the existence of wide resistance behavior to dry and black root rot in *M*. *esculenta* and that it can occur during the breeding process. The results reveal that high disease index (ω) values directly influence genotype survival and plant height, as well as shoot and fresh root yields. Additionally, five significant SNPs were detected for fresh root yield under biotic stress conditions in areas infested with root rot pathogens. However, future analyses and the subsequent validation of the genes described here in larger segregation populations will allow us to illustrate some of the molecular mechanisms for root rot resistance in cassava.

## Supporting information

S1 Table Physical and chemical analysis of the soil in the Embrapa Tabuleiros Costeiros (Umbaúba—SE) experimental field. (DOCX)

S2 Table Overview of the climatological dataset per crop season. (DOCX)

S3 Table Estimated means for survival, disease index (ω), plant height, and shoot and root weights for the extremely susceptible (G3) group formed by cluster analysis. (DOCX)

S4 Table Estimated means for survival, disease index (ω), plant height, and shoot and root weights for the susceptible (G5) group formed by cluster analysis. (DOCX)

S5 Table Estimated means for survival, disease index (ω), plant height, and shoot and root weights for the moderately susceptible (G1) group formed by cluster analysis. (DOCX)

S6 Table Estimated means for survival, disease index (ω), plant height, and shoot and root yields for the moderately resistant (G2) group formed by cluster analysis. (DOCX)

S7 Table Estimated means for survival, disease index (ω), plant height, and shoot and root weights for the resistant (G4) group formed by cluster analysis. (DOCX)
