# Supplementary material for: Genome-wide association study and selection for field resistance to cassava root rot disease and productive traits
Source: PLoS One. 2022 Jun 16;17(6):e0270020. doi: 10.1371/journal.pone.0270020 (PMC9202857; doi:10.1371/journal.pone.0270020)
Supplement: S1 Table — (DOCX) [file pone.0270020.s001.docx]

Supporting information

S1 Table. Physical and chemical analysis of the soil in the Embrapa Tabuleiros Costeiros (Umbaúba - SE) experimental field.

| **Physical Soil Analysis** | | |
| --- | --- | --- |
| **Attributes** |  | **Granulometry (g/kg)** |
| Too coarse sand |  | 35 |
| Coarse sand |  | 147 |
| Medium Sand |  | 189 |
| Fine sand |  | 220 |
| Too Fine sand |  | 83 |
| Total sand |  | 674 |
| Silty |  | 87 |
| Clay |  | 239 |
| Textural class |  | Sandy clay loam |
| **Chemical soil analysis** | | |
| **Features** |  |  |
| **pH H_2_O** |  | 5.14 |
| **P** (mg/dm^3^) |  | 50.8 |
| **K** (mg/ dm^3^) |  | 235 |
| **Ca^2+^** (cmolc/ dm^3^) |  | 3.25 |
| **Mg^2+^** (cmolc/ dm^3^) |  | 2.29 |
| **Al^3+^** (cmolc/ dm^3^) |  | 0 |
| **H + Al** (cmol_c_/ dm^3^) |  | 3.3 |
| **SB** (cmol_c_/ dm^3^) |  | 6.14 |
| **t** (cmol_c_/ dm^3^) |  | 6.14 |
| **T** (cmol_c_/ dm^3^) |  | 9.44 |
| **V** (%) |  | 65 |
| **m** (%) |  | 0 |
| **MO** (dag/kg) |  | 2.69 |
| **P-Rem** (mg/L) |  | 42.1 |
| **Cu** (mg/ dm^3^) |  | 0.74 |
| **Mn** (mg/ dm^3^) |  | 5.8 |
| **Fe (**mg/ dm^3^) |  | 119.4 |
| **Zn (**mg/ dm^3^) |  | 4.15 |
