# Supplementary material for: Genome-wide association study and selection for field resistance to cassava root rot disease and productive traits
Source: PLoS One. 2022 Jun 16;17(6):e0270020. doi: 10.1371/journal.pone.0270020 (PMC9202857; doi:10.1371/journal.pone.0270020)
Supplement: S2 Table — (DOCX) [file pone.0270020.s002.docx]

Supporting information

**S2 Table**. Overview of the climatological dataset per crop season.

| Average | **2014–2015** | **2016­–2017** |
| --- | --- | --- |
| Temperature (°C): | 25 | 25 |
| Relative air humidity (%): | 77.29 | 77.66 |
| Precipitation (mm): | 80.38 | 94.89 |
| Insolation (h): | 191.61 | 195.215 |

*Climate data collected from meteorological stations (INMET).
