# Supplementary material for: Genome-wide association study and selection for field resistance to cassava root rot disease and productive traits
Source: PLoS One. 2022 Jun 16;17(6):e0270020. doi: 10.1371/journal.pone.0270020 (PMC9202857; doi:10.1371/journal.pone.0270020)
Supplement: S3 Table — (DOCX) [file pone.0270020.s003.docx]

Supporting information

S3 Table: Estimated means for survival, disease index (ω), plant height, and shoot and root weights for the extremely susceptible (G3) group formed by cluster analysis.

| **Extremely Susceptible - (G3)** | | | | | |
| --- | --- | --- | --- | --- | --- |
| **Genotype** | **Survival** | **DI (ω)** | **Plant Height** | **Shoot yield** | **Fresh root yield** |
| BGM0598 | 2.06 | 94.94 | 0.00 | 0.00 | 0.00 |
| BGM1027 | 0.83 | 96.12 | 0.00 | 0.00 | 0.00 |
| BGM1345 | 1.85 | 100.00 | 0.00 | 0.00 | 0.00 |
| BGM1365 | 1.76 | 95.26 | 0.00 | 0.00 | 0.00 |
| BGM1464 | 0.91 | 96.00 | 0.00 | 0.00 | 0.00 |
| BGM1832 | 0.91 | 96.00 | 0.00 | 0.00 | 0.00 |
| BGM1865 | 0.91 | 96.00 | 0.00 | 0.00 | 0.00 |
| BGM1867 | 7.56 | 89.59 | 0.00 | 0.00 | 0.00 |
| BGM2020 | 0.91 | 92.08 | 0.00 | 0.00 | 0.00 |
| Minimum | 0.83 | 89.59 | 0.00 | 0.00 | 0.00 |
| Maximum | 7.56 | 100.00 | 0.00 | 0.00 | 0.00 |
| Mean | 1.97 | 95.11 | 0.00 | 0.00 | 0.00 |
