# Supplementary material for: Genome-wide association study and selection for field resistance to cassava root rot disease and productive traits
Source: PLoS One. 2022 Jun 16;17(6):e0270020. doi: 10.1371/journal.pone.0270020 (PMC9202857; doi:10.1371/journal.pone.0270020)
Supplement: S4 Table — (DOCX) [file pone.0270020.s004.docx]

Supporting information

S4 Table. Estimated means for survival, disease index (ω), plant height, and shoot and root weights for the susceptible (G5) group formed by cluster analysis.

| **Susceptible - (G5)** | | | | | |
| --- | --- | --- | --- | --- | --- |
| **Genotype** | **Survival** | **DI (ω)** | **Plant Height** | **Shoot yield** | **Fresh root yield** |
| 9655-02 | 5.67 | 94.04 | 1.68 | 1.15 | 1.59 |
| BGM0083 | 27.64 | 82.23 | 2.01 | 2.41 | 0.26 |
| BGM0148 | 4.64 | 94.82 | 1.45 | 0.42 | 0.06 |
| BGM0190 | 11.52 | 91.02 | 1.57 | 1.35 | 0.02 |
| BGM0205 | 5.07 | 92.98 | 1.49 | 1.19 | 0.06 |
| BGM0249 | 26.75 | 82.77 | 1.81 | 5.85 | 2.19 |
| BGM0279 | 15.18 | 84.87 | 1.91 | 1.50 | 1.86 |
| BGM0376 | 10.43 | 83.89 | 1.52 | 1.34 | 2.23 |
| BGM0408 | 19.94 | 89.80 | 1.52 | 4.88 | 1.64 |
| BGM0465 | 8.56 | 92.30 | 1.72 | 0.77 | 0.55 |
| BGM0511 | 17.97 | 81.97 | 1.93 | 3.76 | 0.18 |
| BGM0512 | 11.52 | 90.54 | 1.87 | 1.48 | 0.05 |
| BGM0541 | 5.67 | 92.63 | 1.82 | 0.63 | 1.55 |
| BGM0544 | 10.43 | 90.12 | 1.86 | 1.05 | 1.66 |
| BGM0550 | 17.97 | 88.11 | 1.86 | 1.64 | 0.05 |
| BGM0552 | 6.60 | 95.84 | 1.51 | 1.67 | 0.49 |
| BGM0563 | 14.74 | 95.63 | 1.43 | 1.65 | 0.07 |
| BGM0579 | 19.94 | 89.80 | 1.66 | 8.07 | 1.67 |
| BGM0664 | 8.30 | 93.45 | 1.45 | 1.88 | 0.15 |
| BGM0706 | 12.48 | 91.85 | 1.89 | 2.09 | 0.56 |
| BGM0776 | 5.67 | 92.08 | 1.76 | 1.22 | 2.30 |
| BGM0822 | 19.94 | 90.12 | 1.39 | 6.06 | 2.22 |
| BGM0868 | 15.18 | 92.08 | 1.67 | 4.61 | 3.19 |
| BGM0876 | 10.43 | 84.44 | 1.75 | 1.13 | 1.55 |
| BGM1138 | 15.18 | 85.21 | 2.00 | 1.51 | 2.61 |
| BGM1163 | 34.21 | 83.25 | 1.69 | 3.97 | 3.20 |
| BGM1174 | 5.67 | 87.49 | 1.89 | 2.75 | 2.96 |
| BGM1185 | 11.50 | 89.08 | 2.19 | 1.32 | 0.54 |
| BGM1202 | 16.40 | 86.58 | 1.84 | 7.01 | 2.35 |
| BGM1318 | 8.30 | 96.10 | 1.54 | 2.45 | 0.05 |
| BGM1324 | 14.74 | 88.33 | 1.81 | 2.69 | 0.07 |
| BGM1344 | 11.52 | 93.08 | 1.84 | 4.70 | 2.34 |
| BGM1440 | 8.30 | 97.72 | 1.51 | 1.96 | 0.05 |
| BGM1482 | 5.67 | 92.08 | 1.32 | 0.59 | 1.55 |
| BGM1590 | 7.07 | 91.01 | 2.04 | 2.09 | 2.39 |
| BGM1942 | 15.18 | 78.94 | 1.49 | 1.35 | 1.57 |
| BGM2061 | 1.75 | 100.00 | 2.05 | 0.30 | 0.01 |
| BGM2066 | 8.30 | 92.10 | 1.30 | 1.72 | 0.17 |
| BGM2083 | 24.41 | 82.03 | 1.68 | 4.55 | 3.56 |
| Fécula Branca | 12.48 | 83.98 | 1.77 | 1.93 | 0.98 |
| BRS Formosa | 14.74 | 84.40 | 1.63 | 3.69 | 1.11 |
| Irará | 5.67 | 93.72 | 1.42 | 0.58 | 1.55 |
| Mani Branca | 13.64 | 92.36 | 1.68 | 3.22 | 0.05 |
| BRS Poti Branca | 8.56 | 91.97 | 1.70 | 4.32 | 1.77 |
|  |  |  |  |  |  |
| Minimum | 1.75 | 78.94 | 1.30 | 0.30 | 0.01 |
| Maximum | 34.21 | 100.00 | 2.19 | 8.07 | 3.56 |
| Mean | 12.63 | 89.70 | 1.70 | 2.51 | 1.25 |
