# Supplementary material for: Genome-wide association study and selection for field resistance to cassava root rot disease and productive traits
Source: PLoS One. 2022 Jun 16;17(6):e0270020. doi: 10.1371/journal.pone.0270020 (PMC9202857; doi:10.1371/journal.pone.0270020)
Supplement: S5 Table — (DOCX) [file pone.0270020.s005.docx]

Supporting information

S5 Table: Estimated means for survival, disease index (ω), plant height, and shoot and root weights for the moderately susceptible (G1) group formed by cluster analysis.

| **Moderately Susceptible - (G1)** | | | | | |
| --- | --- | --- | --- | --- | --- |
| **Genotype** | **Survival** | **DI (ω)** | **Plant Height** | **Shoot yield** | **Fresh root yield** |
| 16_07 | 39.52 | 71.10 | 1.86 | 7.94 | 5.16 |
| 2002_01_01 | 48.48 | 70.24 | 1.74 | 10.30 | 6.13 |
| BGM0022 | 48.07 | 64.77 | 1.71 | 9.76 | 6.08 |
| BGM0133 | 24.41 | 70.34 | 1.74 | 9.78 | 9.11 |
| BGM0140 | 35.08 | 70.76 | 1.51 | 6.04 | 3.39 |
| BGM0163 | 40.18 | 68.40 | 1.95 | 11.45 | 5.20 |
| BGM0217 | 32.04 | 78.21 | 1.75 | 11.69 | 3.76 |
| BGM0254 | 34.09 | 76.47 | 1.65 | 8.32 | 8.20 |
| BGM0319 | 48.48 | 72.37 | 1.97 | 8.48 | 2.80 |
| BGM0425 | 53.24 | 61.13 | 1.81 | 10.04 | 4.44 |
| BGM0428 | 48.48 | 70.89 | 1.67 | 5.78 | 2.06 |
| BGM0517 | 34.09 | 65.13 | 1.68 | 9.02 | 8.60 |
| BGM0543 | 53.24 | 79.25 | 1.76 | 7.65 | 3.92 |
| BGM0785 | 36.59 | 71.58 | 1.69 | 7.11 | 4.67 |
| BGM0877 | 19.94 | 82.15 | 2.37 | 11.70 | 7.88 |
| BGM0930 | 54.62 | 64.80 | 1.81 | 6.47 | 4.43 |
| BGM0945 | 40.53 | 79.91 | 2.21 | 9.71 | 3.82 |
| BGM0960 | 34.09 | 68.07 | 1.75 | 9.44 | 0.27 |
| BGM1137 | 34.21 | 73.40 | 1.98 | 6.33 | 4.78 |
| BGM1175 | 15.18 | 73.82 | 1.94 | 10.85 | 3.53 |
| BGM1287 | 38.86 | 75.65 | 1.72 | 6.57 | 5.98 |
| BGM1387 | 27.64 | 88.57 | 1.55 | 8.89 | 6.41 |
| BGM1495 | 32.08 | 76.13 | 1.95 | 6.75 | 3.46 |
| BGM1521 | 24.41 | 81.69 | 1.69 | 7.06 | 3.76 |
| BGM1523 | 46.10 | 82.08 | 1.64 | 7.44 | 4.90 |
| BGM1719 | 38.97 | 80.88 | 2.04 | 13.86 | 8.03 |
| BGM1884 | 14.74 | 82.71 | 1.97 | 12.37 | 0.02 |
| BGM1957 | 29.45 | 88.57 | 2.04 | 17.44 | 6.89 |
| BGM2048 | 56.65 | 57.47 | 1.46 | 4.82 | 4.65 |
| BGM2054 | 40.53 | 68.06 | 1.92 | 7.94 | 6.17 |
| BGM2055 | 48.23 | 73.85 | 1.77 | 6.61 | 6.04 |
| BGM2060 | 27.64 | 80.01 | 1.53 | 5.50 | 3.24 |
| BRS Caipira | 42.14 | 68.45 | 1.61 | 7.26 | 3.00 |
| BRS Gema de Ovo | 30.86 | 79.93 | 1.74 | 9.42 | 6.80 |
| Salangó | 51.85 | 68.29 | 1.54 | 7.63 | 7.17 |
| BRS Tapioqueira | 30.86 | 74.36 | 1.63 | 8.90 | 4.31 |
| Minimum | 14.74 | 57.47 | 1.46 | 4.82 | 0.02 |
| Maximum | 56.65 | 88.57 | 2.37 | 17.44 | 9.11 |
| Mean | 37.66 | 73.87 | 1.79 | 8.79 | 4.97 |
