# Supplementary material for: Genome-wide association study and selection for field resistance to cassava root rot disease and productive traits
Source: PLoS One. 2022 Jun 16;17(6):e0270020. doi: 10.1371/journal.pone.0270020 (PMC9202857; doi:10.1371/journal.pone.0270020)
Supplement: S6 Table — (DOCX) [file pone.0270020.s006.docx]

Supporting information

S6 Table: Estimated means for survival, disease index (ω), plant height, and shoot and root yields for the moderately resistant (G2) group formed by cluster analysis.

| **Moderately Resistant – (G2)** | | | | | |
| --- | --- | --- | --- | --- | --- |
| **Genotype** | **Survival** | **DI (ω)** | **Plant Height** | **Shoot yield** | **Fresh root yield** |
| BGM0018 | 59.87 | 51.12 | 2.08 | 13.16 | 7.67 |
| BGM0032 | 35.16 | 60.66 | 1.53 | 11.72 | 9.52 |
| BGM0070 | 61.49 | 56.93 | 1.46 | 12.47 | 7.75 |
| BGM0144 | 67.36 | 29.47 | 1.81 | 10.56 | 4.53 |
| BGM0204 | 43.84 | 55.73 | 1.55 | 8.69 | 6.84 |
| BGM0248 | 65.40 | 53.50 | 1.70 | 8.14 | 5.98 |
| BGM0307 | 62.75 | 54.56 | 1.96 | 10.94 | 3.78 |
| BGM0356 | 58.00 | 54.56 | 1.99 | 10.82 | 9.19 |
| BGM0390 | 67.36 | 59.02 | 1.83 | 16.64 | 8.20 |
| BGM0440 | 62.46 | 40.61 | 1.67 | 9.40 | 9.83 |
| BGM0509 | 63.69 | 52.48 | 2.39 | 9.45 | 3.88 |
| BGM0542 | 62.75 | 52.46 | 1.78 | 13.46 | 6.37 |
| BGM0557 | 48.59 | 61.00 | 1.82 | 17.16 | 14.25 |
| BGM0591 | 58.00 | 59.45 | 2.01 | 14.17 | 3.95 |
| BGM0631 | 73.24 | 52.22 | 1.79 | 12.52 | 10.66 |
| BGM0733 | 68.34 | 62.11 | 1.98 | 10.93 | 5.20 |
| BGM0779 | 52.47 | 61.61 | 1.90 | 19.03 | 8.33 |
| BGM0820 | 49.72 | 67.55 | 1.99 | 14.18 | 6.83 |
| BGM0856 | 62.75 | 54.49 | 1.85 | 12.26 | 3.95 |
| BGM1177 | 56.65 | 73.11 | 1.89 | 12.66 | 8.96 |
| BGM1178 | 48.48 | 61.91 | 1.75 | 10.41 | 6.69 |
| BGM1193 | 51.82 | 56.98 | 2.08 | 18.23 | 8.41 |
| BGM1255 | 66.32 | 62.79 | 1.47 | 11.08 | 10.01 |
| BGM1354 | 61.48 | 53.83 | 1.93 | 14.31 | 7.39 |
| BGM1397 | 69.55 | 50.51 | 1.79 | 6.30 | 5.21 |
| BGM1729 | 53.24 | 64.31 | 1.86 | 10.34 | 9.12 |
| BGM1956 | 55.30 | 59.17 | 1.95 | 12.89 | 6.35 |
| BGM2052 | 50.20 | 64.99 | 1.56 | 7.69 | 9.68 |
| BGM2080 | 43.76 | 59.78 | 2.00 | 10.31 | 9.52 |
| BGM2082 | 40.32 | 75.17 | 2.19 | 10.44 | 11.41 |
| Cascuda | 46.98 | 57.46 | 1.58 | 6.39 | 11.33 |
| Cigana Preta | 56.65 | 62.93 | 1.89 | 16.22 | 11.30 |
| BRS Dourada | 66.32 | 43.36 | 1.96 | 13.44 | 5.43 |
| Olho Junto | 43.76 | 65.53 | 1.40 | 10.93 | 15.64 |
| BRS Verdinha | 51.85 | 63.64 | 1.63 | 15.42 | 12.35 |
| Minimum | 35.16 | 29.47 | 1.40 | 6.30 | 3.78 |
| Maximum | 73.24 | 75.17 | 2.39 | 19.03 | 15.64 |
| Mean | 56.74 | 57.57 | 1.83 | 12.08 | 8.16 |
