# Supplementary material for: Genome-wide association study and selection for field resistance to cassava root rot disease and productive traits
Source: PLoS One. 2022 Jun 16;17(6):e0270020. doi: 10.1371/journal.pone.0270020 (PMC9202857; doi:10.1371/journal.pone.0270020)
Supplement: S7 Table — (DOCX) [file pone.0270020.s007.docx]

Supporting information

S7 Table: Estimated means for survival, disease index (ω), plant height, and shoot and root weights for the resistant (G4) group formed by cluster analysis.

| **Resistant - (G4)** | | | | | |
| --- | --- | --- | --- | --- | --- |
| **Genotype** | **Survival** | **DI (ω)** | **Plant Height** | **Shoot yield** | **Fresh root yield** |
| 9975-01 | 79.12 | 37.07 | 1.80 | 17.06 | 11.23 |
| BRS Aipim Brasil | 68.34 | 35.93 | 1.80 | 14.33 | 8.62 |
| BGM0209 | 66.32 | 43.16 | 1.80 | 20.59 | 11.38 |
| BGM0341 | 79.12 | 13.60 | 1.78 | 17.50 | 5.75 |
| BGM0398 | 69.55 | 51.34 | 1.93 | 27.03 | 12.71 |
| BGM0436 | 79.12 | 31.80 | 1.81 | 11.01 | 12.25 |
| BGM0444 | 72.77 | 23.97 | 1.83 | 12.10 | 11.95 |
| BGM0501 | 82.06 | 25.81 | 1.78 | 12.17 | 13.23 |
| BGM0590 | 79.12 | 34.05 | 1.91 | 15.96 | 7.33 |
| BGM0624 | 82.06 | 42.82 | 1.91 | 13.71 | 14.59 |
| BGM0642 | 73.24 | 46.05 | 1.90 | 12.00 | 10.67 |
| BGM0659 | 82.44 | 52.88 | 2.04 | 20.22 | 14.26 |
| BGM0678 | 82.06 | 31.42 | 1.66 | 10.53 | 12.47 |
| BGM0878 | 88.89 | 13.21 | 1.40 | 11.70 | 8.74 |
| BGM1171 | 90.88 | 11.37 | 1.91 | 17.49 | 12.56 |
| BGM1190 | 85.00 | 46.40 | 1.93 | 11.10 | 11.67 |
| BGM1206 | 83.04 | 27.05 | 2.04 | 21.16 | 6.36 |
| BGM1452 | 59.87 | 24.98 | 1.56 | 14.87 | 8.66 |
| BGM1668 | 82.44 | 28.80 | 1.67 | 9.79 | 9.90 |
| BGM2038 | 81.08 | 40.12 | 1.78 | 16.30 | 12.08 |
| BGM2169 | 63.10 | 52.10 | 1.88 | 14.82 | 13.50 |
| Eucalipto | 63.10 | 24.62 | 2.08 | 12.08 | 13.81 |
| IAC90 | 88.89 | 7.80 | 1.57 | 10.12 | 13.61 |
| BRS Kiriris | 61.48 | 48.65 | 2.08 | 18.31 | 17.88 |
| Minimum | 59.87 | 7.80 | 1.40 | 9.79 | 5.75 |
| Maximum | 90.88 | 52.88 | 2.08 | 27.03 | 17.88 |
| Mean | 76.80 | 33.13 | 1.83 | 15.08 | 11.47 |
